# Stability in Vitiligo? What's that?

**DOI:** 10.4103/0974-2077.53100

**Published:** 2009

**Authors:** Koushik Lahiri

**Affiliations:** *Consultant Dermatologist, Apollo Gleneagles Hospitals, Rita Skin Foundation, Kolkata, West Bengal, India*

Since time immemorial, an effective cure for vitiligo has continued to elude the physicians and patients in spite of significant developments in our understanding of the disease process and improvements in management protocols. When the disease becomes refractory to conventional medicinal therapy, transplantation techniques are the only options left to replenish the lost melanocytes. Various surgical modalities and transplantation techniques have evolved during the last few decades.[1–10] For any surgical method to yield good results, proper selection of cases is of paramount importance. The specific criteria for case selection have been well defined.[6] Stability of vitiligo is considered as the most important parameter while opting for any transplantation technique to treat vitiligo.[11]

There are several issues to be considered while defining stability. These include:

- Is stability, area-specific or lesion-specific?
- Defining minimum duration for stability
- Validity of test grafting (TG)
- How long does stability last?
- Concept of cellular stability

Even after recognizing the concept of stability and after three decades of experience in vitiligo surgery, it is paradoxical to note that consensus does not exist regarding the optimal required period of stability. The total lack of agreement amongst different workers can be glaring, as seen in Table 1.[12–18] Recently, in their consensus recommendations, the IADVL Task Force for standard guidelines of care for dermatosurgical procedures attempted to provide a clear definition of stability as *‘a patient reporting no new lesions, no progression of existing lesions, and absence of Koebner phenomenon during the past one year’*.[19]

**Table 1:** Minimum period of stability in different studies

Author	Year	Period of stability
Das SS, Pasricha JS[12]	1992	4 months
Boersma BR, Westerhof W[13]	1995	6 months
Jha AK, Pandey SS, Shukla V K[14]	1992	1 year
Savant SS[15]	1992	2 years
Falabella R[16]	2003	2 years
Falabella R[17]	1995	2 years
Falabella R[18]	1992	3 years

Stability may be lesion-specific in many patients. Due to poorly understood reasons, simultaneous depigmentation and repigmentation have been noticed in different areas of the same patient.[20] Variable results have been obtained over the donor site and recipient site of the same patient.[20–22] This area-based variable status of stability is neither related to the conventional refractory behavior of vitiligo, typically seen in the so-called ‘resistant’ anatomical sites such as palms, soles, lips, nipples, areola, glans penis and bony prominences nor is it related to the type of vitiligo such as unilateral (segmental/focal) or bilateral (symmetric, vulgaris, or generalized).

With the backdrop of a persistent inappropriateness about the minimal period of stability, an attempt was made for the first time by Falabella in 1995 to define stability by introducing minigrafting test.[22] The objective of this test was to serve several purposes:

- Establishing the stability of the depigmenting process
- Determining a means by which patients could be selected
- Identifying patients who may respond to pigment cell transplantation
- Anticipating the response to surgical repair

This test grafting (TG) has been found to be a more reliable exercise than the arbitrarily defined period of stability alone.[22–24] Over the years, the validity of this ‘test’ has been vindicated and acknowledged as an important tool for detecting stable vitiligo, which anticipates the repigmentation success in vitiligo when surgery is chosen as a therapeutic option.

However, the concept of TG too has been subjected to skepticism. In the original article, no mention was made about the donor site.[13] It has been suggested that the behavior of the donor area too should be taken into consideration, concurrently along with the behavior of the mini-grafts in the recipient site. That might have added more comprehensive idea about the status of stability.[22]

But doubts have been expressed about overdependence on TG *per se* in view of the following possible fallacies.[21,24]

- TG positivity does not necessarily guarantee a favorable outcome in all cases even after perfect graft take-up.
- Encouraging results were obtained even after grafting in some TG-negative cases.
- Simultaneous donor site repigmentation and depigmentation of grafts at the recipient site or donor site depigmentation with complete repigmentation of the recipient area have been observed.

Juhlin echoed this apprehension and observed that, “*…neither the history nor test graft is a complete help to obtain good effect. It seems we have to wait until the method of identification and isolation of skin-homing melanocyte-specific cytotoxic T lymphocytes is available*”.[25] Falabella himself expressed his doubts about the comprehensiveness of the minigraft test. He observed, ‘*even when the minigrafting test is positive, ‘depigmenting activity’ may still be present and prevent satisfactory repigmentation*’.[26]

More recently, Njoo *et al.,* explored the association between the experimentally induced Koebner phenomenon (KP-e) and the Kobner phenomenon by history (KP-h), disease activity, and therapeutic responsiveness in vitiligo vulgaris.[11] In their article, they proposed to measure disease activity on a 6-point scale from −1 to +4 (vitiligo disease activity score, VIDA) and therapy-induced repigmentation grade. But despite the more comprehensive nature of the scoring system, VIDA scoring system was found to lack application potential. The authors have observed and concluded that,

- The VIDA score did not always predict a positive KP-e
- The KP-e may function well as a clinical factor to assess present disease activity and may also predict the responsiveness to fluticasone propionate plus UV-A therapy but not to UV-B (311 nm) therapy.

In this context, it can be concluded that overdependence on KP or TG may be sometimes misleading in this enigmatic disease. Because what these two reveal is the apparent clinical stability only and that may not be the true reflection of the stability status of the disease at the cellular level.[24]

Depigmentation of grafts was documented in herpes labialis induced lip leucoderma, which was clinically stable.[27,28]

Another important indicator is the longevity of the stability status of vitiligo. In other words, how long would the disease remain stable, after achieving “stability”? It is hard to predict how long the disease will remain stable and also to envisage when it will start to become unstable later. Repigmentation has been successfully induced in previous graft failure cases by NB-UVB (311nm) phototherapy.[29] It was also proposed that NB-UVB might have played the pivotal role of stabilizing the disease by promoting apoptosis of the perilesional and circulating melanocyte-specific cytotoxic CD8 cells.[30,31]

A recent study proposed one year as the “period of stability of repigmentation” (as opposed to period of inactivity discussed so far). That means, to qualify as ‘stable’, the repigmentated status must be maintained for a period of not less than one year.[32]

## SO, WHERE DO WE STAND?

It is obvious that before any attempt to predict the outcome of any vitiligo surgery, assessment of ‘cellular’ stability is of paramount importance and the cellular parameters should also be taken into consideration, along with the clinical stability. When these ultarstructural investigation facilities become available, it may be possible to clearly define stability not only on clinical grounds but also electron microscopic and histoenzymologic analysis of the perilesional and nonlesional skin of vitiligo patients. Probably, some growth factors which are responsible for both mitogenic and melanogenic stimulation of melanocytes may need to be taken into account. Some serological test(s) may enable us to measure these growth factors.

However, we should not refrain from chosing the surgical option till those unexplored areas are charted and the cellular parameters become available; rather we should continue surgical intervention based on logical application of our clinical knowledge. Clinical criteria based on different factors such as duration of stability, activity of disease, KP and TG should all be the criteria to help us choose a suitable patient for surgery. And as in any surgical procedure, the situation of a given patient and his/her needs should always be given the paramount importance in decision-making.
